# Persistent nipple discharge after nipple-sparing mastectomy secondary to unincorporated AlloDerm: a case report

**DOI:** 10.1186/s13256-020-02476-9

**Published:** 2020-09-24

**Authors:** Avra S. Laarakker, Audrey Rich, Jeffrey Wu, Stephanie Fine

**Affiliations:** 1 Division of Plastic, Reconstructive and Burn Surgery, MSC10 5610, 1 University of New Mexico, Albuquerque, NM 87131 USA; 2 University of New Mexico School of Medicine, Albuquerque, NM USA; 3 Division of Surgical Oncology, University of New Mexico, Albuquerque, NM USA

**Keywords:** Nipple discharge, Nipple-sparing mastectomy, Seroma, AlloDerm, Unincorporated AlloDerm, Case report

## Abstract

**Background:**

Rates of nipple-sparing mastectomies have increased over the past decade. In 2017, acellular dermal matrix was used in 56% of breast reconstructive procedures, with complication rates similar to operations without AlloDerm. Although persistent nipple discharge after nipple-sparing mastectomy is a rare event, it has been described in the literature. Other authors have described evaluation and treatment on a case-by-case basis. To the best of our knowledge, this is the first case report to describe a persistent unilateral discharge after multiple operative revisions and to provide an algorithmic approach to workup and treatment.

**Case presentation:**

We present a case of a 29-year-old Hispanic woman with *BRCA1* mutation who underwent a prophylactic bilateral nipple-sparing mastectomy with immediate reconstruction using AlloDerm. The year following her operation, the patient underwent two surgical revisions, one for implant rippling and one for asymmetry. Six months after her second revision, she presented to our hospital with a capsular contracture and unilateral clear nipple discharge. Her breast ultrasound showed dilated subareolar ducts and a suspicious mass. Magnetic resonance imaging identified a benign-appearing, rim-enhancing fluid collection. She underwent a third revision. One year later, she returned to our clinic with bloody nipple discharge, erythematous skin changes, and a palpable breast lump. Her surgical biopsy showed a fold in AlloDerm and chronic inflammatory changes. She continued experiencing discharge and opted for nipple excision. During the operation, a lacrimal probe demonstrated a direct connection between the discharging external duct and a seroma associated with an area of unincorporated AlloDerm. The section of unincorporated AlloDerm was excised, and no evidence of malignancy was identified. Ten months later, the patient remained symptom-free and had progressed to placement of final silicone implants.

**Conclusions:**

To the best of our knowledge, this is the first case report to describe a nongravid patient with persistent unilateral sanguineous nipple discharge after multiple operative revisions. A visible communication between the draining duct and a seroma associated with unincorporated AlloDerm was ultimately identified. We present a clinical algorithm for patients with nipple discharge after nipple-sparing mastectomy.

## Background

Rates of nipple-sparing mastectomy (NSM) are increasing, and although persistent nipple discharge after NSM is a rare event, this is the first case report, to the best of our knowledge, to describe a nonpregnant patient with persistent unilateral sanguineous nipple discharge after multiple revisional operations. A visible communication between the draining duct and a seroma associated with unincorporated AlloDerm (Allergan, Madison, NJ, USA) was intraoperatively identified as the cause of the persistent discharge.

## Case presentation

Our patient first presented to our hospital as a 29-year-old, nonpregnant, G4P2-0-2-2 Hispanic woman with a *BRCA1* deleterious gene mutation who underwent prophylactic bilateral NSM and immediate direct-to-implant subpectoral reconstruction using an AlloDerm sling. Three months from the initial operation, the patient underwent breast reconstruction revision with bilateral implant replacement, bilateral superior and lateral capsulotomies, and bilateral fat grafting due to implant rippling with inadequate upper pole projection. She underwent a second revision due to asymmetry several months later, which included a right inferior capsulorrhaphy, bilateral fat grafting, and right breast mastopexy.

Six months later, she noticed left-sided clear, yellow nipple discharge from a single duct. The discharge was reproducible with firm pressure. A grade III capsular contracture was present without erythema or evidence of skin lesions. Her breast ultrasound showed unilateral dilated subareolar ducts and a suspicious 1.2-cm heterogeneous mass. Breast magnetic resonance imaging (MRI) identified a thin, circumferential rim-enhancing fluid collection associated with the left implant, without nipple enhancement, and confirmed the heterogeneous mass to be an area of fat necrosis. She underwent revisional excision of residual subareolar tissue, left breast capsulectomy, implant exchange using AlloDerm, and bilateral breast fat grafting. Pathology showed benign breast ducts and lobules without proliferative changes, atypia, or neoplasm.

**Fig. 1:**
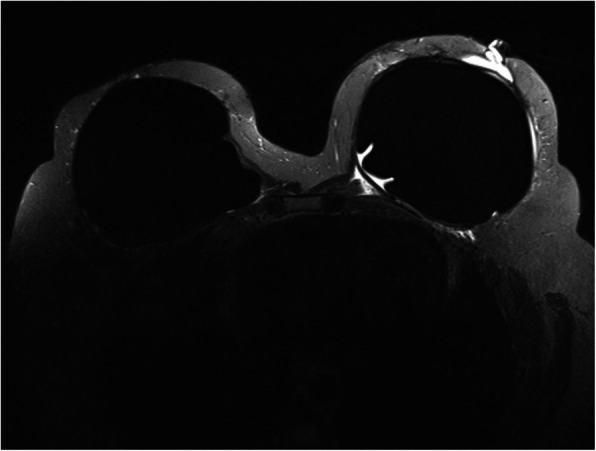
Axial T2-weighted image of the left breast with fat suppression after contrast obtained showing a recurrent fluid collection extending into the nipple ducts

One year later, she returned to our hospital with complaints of left bloody nipple discharge, erythematous skin changes noncontiguous with the nipple–areola complex (NAC), and a left lateral breast lump. An MRI scan again noted the same benign-appearing, small-volume, peri-implant fluid collection, but now with visible extension to the nipple ducts (Fig. 1). After two failed courses of antibiotics, operative evaluation was performed to biopsy the erythematous skin and assess the lateral breast irregularity. The skin biopsy showed chronic inflammatory changes, and the lateral breast mass was a fold in the AlloDerm.

**Fig. 2:**
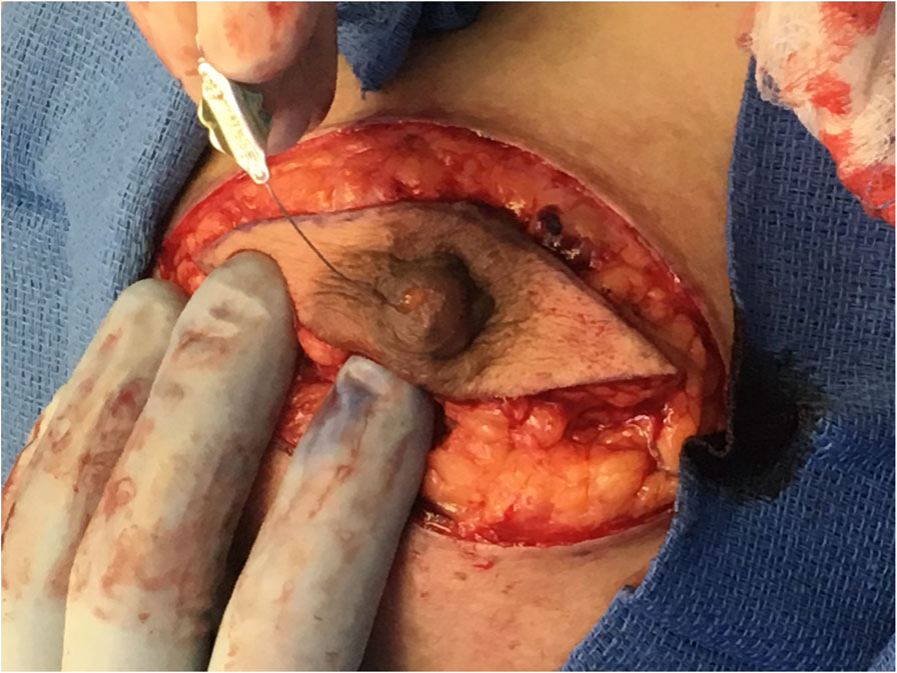
Lacrimal probe placed in the duct with expressible nipple discharge demonstrating direct connection between the leaking duct and a seroma associated with an area of unincorporated AlloDerm

Over the next 6 months, she continued to experience left nipple discharge that left half-dollar–sized stains on her clothing, usually following exercise or warm showers. A decision was made to return to the operating room for bilateral nipple excision (bilateral for symmetry) and conversion of subpectoral implants to AlloDerm-wrapped prepectoral implants. A lacrimal probe demonstrated a direct connection between the discharging external duct and a seroma associated with an area of unincorporated AlloDerm. No pathologic evidence of malignancy was found in either the excised nipple or other submitted tissues (Fig. 2).

Ten months following the removal of unincorporated AlloDerm, the patient remained free of signs or symptoms of a recurrent seroma and had progressed to placement of final silicone implants.

## Discussion

Rates of NSM have increased over the past decade, with patients seeking the benefits of this procedure for both therapeutic and prophylactic indications. In 2017, members of the American Society of Plastic Surgeons used acellular dermal matrix in 59,774 of 106,295 breast reconstructive surgeries (56% of procedures) [1]. A meta-analysis by Heidemann and colleagues reported complication rates of NSM with the use of AlloDerm Ready To Use (AlloDerm RTU; LifeCell, Branchburg, NJ, USA) as 9% necrosis or ischemia, 4% NAC necrosis or ischemia, 12% infection, 1% hematoma, 5% seroma, 4% explantation, and 9% unplanned return to the operating room. These rates are similar to that of NSM with reconstruction without the use of AlloDerm [2].

Despite its widespread use, unincorporated AlloDerm is a rare event. Animal research demonstrates complete integration of the graft at 12 weeks with evidence of vascularization seen on both sides [3]. Gabriel and colleagues described a rate of 1.5% for unincorporated graft material in a series of 68 patients (116 breasts) whose AlloDerm RTU grafts were biopsied during routine expander/implant exchange [4].

Persistent nipple discharge in the setting of a prior NSM in postpartum and nonpregnant patients has been described. Tang and colleagues reviewed 1620 NSM patients and identified 27 women who carried successful term pregnancies following an NSM operation. Records show that 6 (22%) of 27 experienced nipple discharge following delivery; 5 women described the discharge as “milky” and 1 as “clear.” All six women reported that discharge stopped after cessation of either breastfeeding (from the contralateral side) or cessation of fertility treatments. There was even distribution of indications in this small group, both risk reduction and malignancy. Of the remaining 1593 patients who did not become pregnant after their NSM, 4 reported clear discharge (0.25%), 2 of whom had acellular dermal matrix slings in place. One patient opted for complete nipple excision, which resolved the issue, and the other opted just for excision of the nipple’s discharging orifice. This failed to correct the discharge, and the patient declined further intervention. Mean follow-up was 54 months, and all were without evidence of a new or recurrent malignancy. The authors describe “extensive evaluation” of the early patients, although they do not provide full details. Interestingly, in this group of ten patients who experienced nipple discharge, only one nipple was resected, revealing normal histology [5].

Orzalesi and associates documented the only other case of nipple discharge following NSM identified in the literature. They described a 40-year-old woman who developed serosanguinous nipple discharge 2 years after the procedure. Discharge cytology results were suspicious for malignancy, but galactography suggested benign duct ectasia. The patient underwent a selective duct resection that resolved the discharge and allowed preservation of the NAC. Final pathology confirmed columnar cell metaplasia with fibrocystic mastopathy and necrosis [6].

There is literature to support the idea that nipple discharge after breast surgery can be linked to the presence of a seroma. Ramirez-Hernandez *et al.* documented two instances of spontaneous nipple discharge following lumpectomy. Using galactography, they identified the source of discharge as a fistulous connection between native ducts and a postlumpectomy seroma [7]. Koretz and Strano described a similar event involving a 37-year-old woman who developed spontaneous bloody nipple discharge 1 month after a lumpectomy with axillary lymph node dissection. Communication with the peripheral surgical site seroma was identified by galactography and confirmed by ultrasound. The discharge was self-limited and required no surgical intervention [8].

Our patient first noticed nipple discharge after the third breast operation, approximately 12 months after her initial surgery. She described increases in discharge after activity and warm showers, similar to other patients described in the literature. Despite the lack of malignant enhancement by MRI, the evolution of the discharge character from serous to bloody, and an underlying *BRCA1* mutation, elevated our concern for a missed diagnosis of cancer. We found no evidence of cancer. Our patient had several features in common with the other reported cases: normal appearance of the NAC, increase in discharge volume following activity or warm showers, and a lack of specific imaging features suggestive of malignancy.

**Fig. 3:**
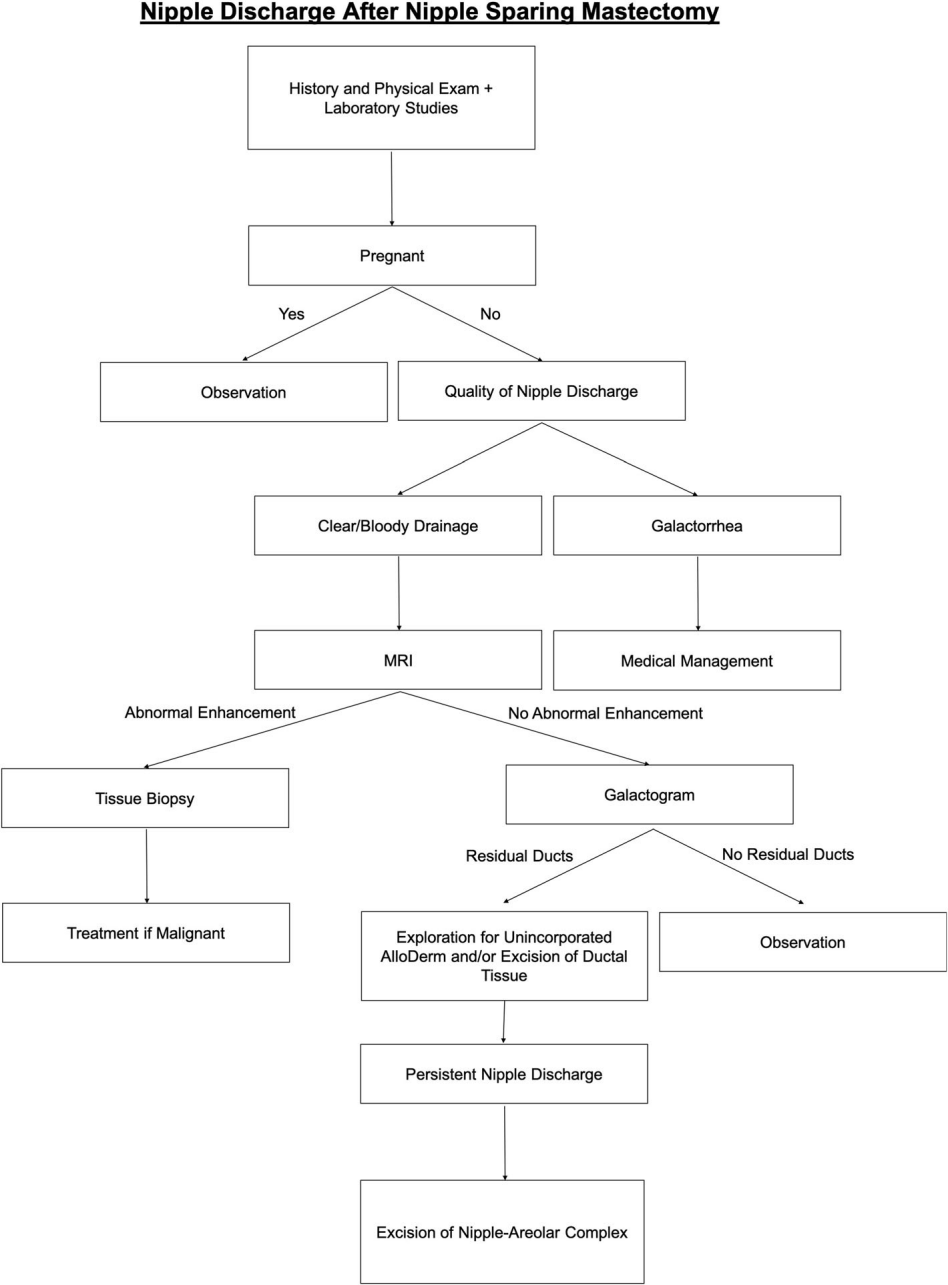
Nipple discharge after nipple-sparing mastectomy: an algorithm for workup of a patient with nipple discharge follow nipple-sparing mastectomy

On the basis of our literature review as well as our experience, we suggest the following algorithm when managing a patient with nipple discharge following NSM (Fig. 3). Initial evaluation includes taking a relevant history, conducting a physical examination, establishing a baseline appearance of the NAC, and performing the following laboratory tests: β-human chorionic gonadotropin, prolactin, thyroid-stimulating hormone, and blood urea nitrogen/creatinine. If the patient is pregnant, observation is recommended because the discharge will likely resolve with cessation of lactation stimulus. If the patient is not pregnant and has galactorrhea, medical management can be considered. In a nonpregnant patient with clear or bloody discharge, we recommend obtaining an MRI scan. If MRI reveals abnormal enhancement, especially in a patient with prior malignancy, a tissue biopsy is warranted. If there is no abnormal enhancement or there is evidence of a seroma surrounding the AlloDerm-wrapped implant, observation is reasonable because there is low risk of recurrent or residual malignancy. A galactogram may establish a residual duct pattern or identify an intraductal lesion or a connection with a previously identified seroma, especially in the setting of persistent or concerning discharge. If the discharge is persistent or problematic to the patient, one can consider further excision or conversion to a non-NSM. Routine breast screening throughout this time is still recommended for the intact breast.

Although the University of New Mexico Institutional Review Board does not require approval of retrospective descriptive studies consisting of three or fewer cases, the patient gave us direct written informed consent to publish our experience and use the accompanying images.

## Conclusion

Nipple discharge following NSM is a rare event. Our review of the literature did not reveal an underlying malignancy in the limited number of case reports that specifically describe normal appearance of the discharging nipple with or without normal imaging. Our experience demonstrates that ductal communication with a seroma surrounding unincorporated AlloDerm can be a cause of nipple discharge. Furthermore, we suggest a treatment algorithm based on a review of the literature and our experience.

## Data Availability

All data generated or analyzed during this study are included in this published article.

## Abbreviations

MRI: Magnetic resonance imaging
NAC: Nipple–areola complex
NSM: Nipple-sparing mastectomy
